# Grand challenge in Biomaterials-wound healing

**DOI:** 10.1093/rb/rbw015

**Published:** 2016-03-08

**Authors:** Joseph C. Salamone, Ann Beal Salamone, Katelyn Swindle-Reilly, Kelly Xiaoyu-Chen Leung, Rebecca E. McMahon

**Affiliations:** Rochal Industries LLC, 12719 Cranes Mill, San Antonio, TX, 78230, USA

**Keywords:** non-stinging liquid adhesive bandages

## Abstract

Providing improved health care for wound, burn and surgical patients is a major goal for enhancing patient well-being, in addition to reducing the high cost of current health care treatment. The introduction of new and novel biomaterials and biomedical devices is anticipated to have a profound effect on the future improvement of many deleterious health issues. This publication will discuss the development of novel non-stinging liquid adhesive bandages in healthcare applications developed by Rochal Industries. The scientists/engineers at Rochal have participated in commercializing products in the field of ophthalmology, including rigid gas permeable contact lenses, soft hydrogel contact lenses, silicone hydrogel contact lenses, contact lens care solutions and cleaners, intraocular lens materials, intraocular controlled drug delivery, topical/intraocular anesthesia, and in the field of wound care, as non-stinging, spray-on liquid bandages to protect skin from moisture and body fluids and medical adhesive-related skin injuries. Current areas of entrepreneurial activity at Rochal Industries pertain to the development of new classes of biomaterials for wound healing, primarily in regard to microbial infection, chronic wound care, burn injuries and surgical procedures, with emphasis on innovation in product creation, which include cell-compatible substrates/scaffolds for wound healing, antimicrobial materials for opportunistic pathogens and biofilm reduction, necrotic wound debridement, scar remediation, treatment of diabetic ulcers, amelioration of pressure ulcers, amelioration of neuropathic pain and adjuvants for skin tissue substitutes.

A major area of Rochal’s activities relates generally to biological applications of non-stinging liquid adhesive materials that are useful for protecting surfaces, such as medical devices and biological surfaces, including skin and mucous membranes, from pressure, shear and friction, and where antimicrobial compositions may also protect against microorganisms.

Liquid bandages have become popular in recent years because of their ease of use in topical applications for protecting skin and/or for preventing damage to skin by forming conformal, oxygen and water-vapor permeable, transparent, topical coatings after solvent evaporation. An example of this technology is exemplified by 3M Cavilon No Sting Barrier Film.

Liquid bandages are used to protect fragile skin from damage caused by incontinence, ostomy leakage, adhesive tape removal and infusion sites areas, as well as periwound skin protection.

For non-stinging applications, the liquid bandages have been obtained from polymer coatings that are delivered from evaporation of soluble solutions in volatile, non-stinging solvents (to skin and open wounds), such as hexamethyldisiloxane (HMDS) and isooctane (2,2,4-trimethylpentane); formulations may also be delivered from stinging alcohol-based solvents, such as isopropanol and ethanol, for inherent antimicrobial character and the potential to solubilize many antimicrobial agents.

Alkylsiloxysiloxane-containing hydrophobic polymers have been prepared in liquid polydimethylsiloxanes (U.S. Pat. 5,103 812 and U.S. Pat. 4,987 893) to provide non-stinging, non-irritating coating materials that allow body fluid evaporation and oxygen permeability while protecting the body surface from further contamination and desiccation. In another variation, alkylsiloxysiloxane-containing polymers were mixed with 2,2,4-trimethylpentane to provide similar non-stinging coating properties (U.S. Pat. 6,383 502). Such hydrophobic coatings may have the common disadvantages of loss of adhesion toward hydrated surfaces, loss of adhesion in higher flexibility areas such as knuckles or knees, and being adherent to themselves or other objects, at room temperature or body temperature. The primary alkylsiloxysilane monomer for these polymer coatings has been 3-methacryloyloxypropyltris(trimethylsiloxy)silane (TRIS, also known as 3-[tris(trimethylsiloxoy)silyl]propyl methacrylate).

A major difficulty with such hydrophobic polymer coatings that had not been addressed was their potential adhesion to undergarments, bed clothes, sheets, blankets, tubing, medical devices and dressings, as well as attachment to more than one portion of one’s body, such as a folded arm, a folded leg, under a chin, or under a breast. When heated to body temperature, such coatings can adhere to skin and to surfaces with which the polymer-coated skin is in contact. In combination with external body temperature (33–37^°^C) and pressure applied to different portions of the body, the air surface of the polymer coating can fold and adhere to itself, causing discomfort. In particular, if an individual is confined to bed and has limited mobility, the pressure of lying in one place for extended periods of time, combined with high shear forces when the person is raised or lowered on the bed, sliding against the bed sheets, the liquid bandage can adhere to the bed sheets and hence cause damage, particularly to fragile skin and wounds. This feature is a result of sustained pressure, friction and shear. A similar problem can occur with medical devices where friction, pressure and shear forces can cause discomfort to a patient with movement.

Cyanoacrylates have also found use as liquid adhesive bandages, particularly n-butyl and 2-octyl cyanoacrylates (U.S. Pat. 6,183 593; U.S. Pat. 6,143 805). These monomers provide quick polymer film formation and are especially useful for closing thin wounds, such as those created by paper or razor cuts. Wounds that are in high flex areas are not suitable for treatment with cyanoacrylates as they tend to increase scarring, if well adhered, or to delaminate quickly, if not well adhered due to their intrinsic brittleness.

Hydrogel liquid bandages have been available for human, veterinary and device use, such as JUC Liquid Bandage Spray from NMS Technologies that is reported to form a positively charged antimicrobial coating. Poly(N-vinylpyrrolidone) water-based liquid bandages have also been available for veterinary use. Whereas water-based liquid bandages are normally less traumatic when applied to an open wound than application of an organic solvent (such as ethanol, isopropanol, ethyl acetate, acetone), the drying time of a polymer film deposited from water can be long, and a polymer coating may be difficult to form in a desired location because of flow of the formulation away from the wound, or removal before drying has occurred.

An advanced liquid adhesive bandage developed by Rochal Industries provides a liquid, amphiphilic, siloxysilane polymer-containing coating material, with or without an antimicrobial agent, that can act as a bandage or coating on skin, on a device or on a dressing to prevent damage to wounds, skin or mucosal tissue resulting from applied pressure, friction and shear forces. This unique polymer is soluble in polar and non-polar solvents and when cast and dried yields an adherent polymer coating on a surface, particularly to a skin surface, wherein when the polymer coating is folded against itself or placed against another material, the surface of the coating does not adhere, while the bottom of the coating remains attached to the original surface. This phenomenon has not previously been observed in any hydrophobic (U.S. Pat. 5,103 812) or amphiphilic (U.S. Pat. 7,795 326) siloxysilane-based liquid bandage formulation, but has been observed in certain cyanoacrylate monomer formulations (U.S. Pat. 7,641 893).

The amphiphilic polymer coating is applied in liquid form and air dried at room or body temperature on a biological surface or medical device to form an adherent, water-insoluble, water-vapor permeable, oxygen permeable, conformal, non-biodegradable, solid, clear, protective film. Application of the coating solution can be by spraying, wiping, dipping, painting, casting, brushing and by aerosol propellants, or by other conventional coating methods, to coat a surface or device. The liquid adhesive materials are useful for protecting surfaces, such as biological surfaces, including skin and mucous membranes, and medical devices. The cast and dried amphiphilic polymer coatings are insoluble in water, but the coatings allow for water vapor transmission and oxygen permeability, primarily by the siloxysilane monomer component. Siloxysilane-containing polymers are noted for their water-vapor permeability and their gas permeability. Such polymers have been used in contact lens materials as crosslinked soft silicone hydrogels or as crosslinked rigid gas permeable materials because of their high oxygen permeability (U.S. Pat. 4,152 508; U.S. Pat. 7,795 326; U.S. Pat. 8,415 404) and as liquid adhesive bandages because of their oxygen permeability, water-vapor transmission (U.S. Pat. 7,795 326) and transparency.

When the amphiphilic siloxysilane polymer coating material is applied to a biological surface, such as skin, it has the surprising property of not being adherent to itself when folded, or when placed against another object, such as clothing or bed sheets and blankets. This feature is highly beneficial in the reduction of friction when stress or pressure is applied to a wound or potential wound environment.

In some formulations, hydrophilic monomers can be included in the amphiphilic polymer provided that they do not cause adherence of two films together, or adherence of a polymer film to another substrate. Such formulations can also be cast from alcohol solutions.

The liquid, polymer-containing coating materials can be applied over a temperature range of −10°C to + 45°C when applied to skin, wounds, nails, mucous membranes, medical devices and other inanimate objects to form films that dry rapidly. In particular, it is a property of the liquid, polymer-containing coating materials that once the coating material is applied at room temperature, the adherent coating can form in less than 1 minute.

These amphiphilic siloxysilane polymers are semi-occlusive with moisture vapor transmission rates between 300 and 700 g/m^2^/24 h. Semi-occlusive wound dressings provide a preferred wound environment where the wound neither desiccates nor becomes macerated from physiological fluids. The oxygen transmissibility of these polymers (2.3–2.6 ml/(m^2^/min)) is approximately four times higher than the skin’s transmissibility (0.5 ml/(m^2^/min)). Therefore, oxygen is available for wound healing.

When combined with an antimicrobial agent, such polymer coatings provide additional protection to wounds by combining the protective coating of the polymer film with its intimately conformal adhesive properties, its semi-occlusive water-vapor properties, its oxygen permeability, its low surface friction, and its ability to prevent the ingress and growth of microorganisms. Such polymer coatings have great promise in facilitating wound healing.

## Conflict of interest statement

All authors of this manuscript have been employees of Rochal Industries LLC at the time this study was conducted. Ann Beal Salamone, (President and Director) and Joseph C. Salamone (Chief Science Officer) are founders and shareholders of Rochal Industries LLC. Katelyn E. Reilly and Kelly Xiaoyu-Chen Leung are Senior Scientists and shareholders of Rochal Industries LLC, with Katelyn E. Reilly now in the Department of Biomedical Engineering of Ohio State University. Rebecca E. McMahon is a Senior Scientist.

